# Foliar thiourea and potassium nitrate enhance physiological performance, antioxidant defense, and yield of heat-stressed wheat under field conditions

**DOI:** 10.3389/fpls.2026.1766062

**Published:** 2026-04-10

**Authors:** Yahya Alzahrani, Hameed Alsamadany, Zahid H. Shah

**Affiliations:** 1 Department of Biological Sciences, Faculty of Science, King Abdulaziz University, Jeddah, Saudi Arabia; 2 College of Agriculture, Jilin Agricultural University, Changchun, China

**Keywords:** gene regulation, multivariate analysis, oxidative stress, stress tolerance, trait association

## Abstract

Terminal heat stress negatively affects wheat by disrupting physiological, biochemical, and agronomic traits. This study examined the effects of thiourea (50 µM) and KNO_3_ (0.5%), applied individually or together, on a heat-sensitive wheat genotype (HS-240) grown under field conditions. Treatments were: T_0_ (control), T_1_ (thiourea), T_2_ (KNO_3_), and T_3_ (thiourea + KNO_3_). The combined treatment (T_3_) improved key physiological traits, including photosynthesis rate (14.2 vs. 8.8 µmol CO_2_ m^-^² s^-^¹), relative water content (83% vs. 65%), and chlorophyll stability (SPAD 42 vs. 28) compared to control. Biochemical defenses were also enhanced, with higher levels of proline, soluble sugars, total phenolics, and stronger antioxidant enzyme activities (SOD, CAT, POD, APX). Oxidative damage decreased, as indicated by lower electrolyte leakage and MDA, and canopy temperature depression increased (2.5 °C → 9.0 °C). These physiological and biochemical improvements translated into better crop performance, with higher grain yield (16 g vs. 12 g per plant), thousand-grain weight (42 g vs. 34 g), and harvest index (49%). Molecular analysis showed that T_3_ strongly upregulated genes involved in stress tolerance, antioxidants, and yield regulation (*TaHSP17, TaHSP90, TaSOD, TaCAT, TaAPX, TaP5CS, TaDREB2, TaSUS, TaGW2, TaCKX2*). Multivariate analyses (correlation, PCA, heatmap) confirmed positive associations between photosynthesis, antioxidant activity, and yield traits. Overall, the combined application of thiourea and KNO_3_ enhances wheat heat tolerance through integrated physiological, biochemical, and molecular mechanisms, offering a practical and cost-effective strategy to sustain wheat productivity under high-temperature stress.

## 1 Introduction

Wheat (*Triticum aestivum* L.) is an important staple crop, providing approximately 20% of the world food calories and protein (FAO., 2023). Its production is highly threatened by climatic stresses, with heat stress being one of the most important abiotic factors. Even short spells of high temperature during booting, heading or grain filling stages can damage photosynthesis (Pn), disrupt assimilate translocation, and reduce yield including thousand-grain weight and grain number (Barnabás et al., 2008; Raza et al., 2019). It is projected that each 1 °C rise above the optimum temperature can decrease global wheat productivity by about 6 to 7% (Zhang et al., 2018).

Plants have natural tendency to trigger a range of defense mechanisms against heat stress, including antioxidant systems, osmolyte concentration, and physiological regulation. However, these natural responses are often insufficient during severe and long duration of heat stress (Wahid et al., 2007). Therefore, supplementary agronomic approaches enhancing these defense mechanisms have become increasingly important (Farooq et al., 2011). Among such approaches, the application of osmoprotectants has attracted attention as a simple, cost effective, and environmentally safe field practice (Yadav et al., 2022).

Thiourea (TU), a sulfhydral compound, has been reported to enhance wheat stress tolerance by improving RWC, stabilizing chlorophyll, maintaining Pn, and triggering antioxidant defenses (Hemanth et al., 2019). On the other hand, TU reduces malondialdehyde (MDA) and electrolyte leakage (EL) through enhancing the proline, soluble sugars (SS), and antioxidant enzymes under heat and drought stresses (Ishfaq et al., 2024). In the same way KNO_3_ exhibits a significant role in stress alleviation by supplying both potassium and nitrate ions, which are essential for stomatal conductance (Gs), osmotic adjustments and antioxidant enzyme activation (Marschner, 2012). Besides, foliar KNO_3_ supplementation enhances canopy temperature depression (CTD), improves chlorophyll stability, supports tillering, triggers assimilate translocation and increase wheat during heat stress at terminal growth stage (Hemanth et al., 2019). The KNO_3_ and TU may provide coactive benefits on integrated application by synergistically improving antioxidant activity, proline concentration, canopy cooling, osmoprotection and agronomic production (Singh et al., 2025). Till note, few studies have been reported under field conditions, linking physiological, biochemical and molecular indicators of heat stress tolerance with agronomic yield, particularly in high yielding susceptible cultivar during terminal growth stages using exogenous osmoprotectants. In addition to physiochemical and agronomic markers, the expression of key heat-responsive genes plays an important role in imparting wheat tolerance against terminal heat stress. Genes such as heat shock proteins (*TaHSP17, TaHSP90*) sustain proteins and photosystems under enhanced temperature (Wang Y. et al., 2023). Besides, transcription factors like *TaDREB2* modulate Gs and RWC (Yadav et al., 2022), while antioxidant genes *TaSOD, TaCAT* and *TaAPX* protect the cells against oxidative damage caused by heat stress (Fu et al., 2022). Li et al. (2023) described the important role of *TaP5CS* in proline biosynthesis and osmotic homeostasis. Moreover, the upregulation of yield-regulators such as TaGW2*, TaCKX2*, and *TaSUS* due to osmoprotectants sustain grain size, spike fertility, and assimilate translocation during heat stress at terminal growth stages (Zhang et al., 2021). In this context, integrating physiological, biochemical, and agronomic traits with the expression of key gene regulators provides a comprehensive understanding of wheat’s adaptive mechanisms under terminal heat stress. Therefore, the present study aimed to elucidate the effects of individual and combined foliar applications of thiourea (TU) and KNO_3_ on physiological, biochemical, and yield-related traits of a heat-sensitive wheat cultivar under field heat stress. Additionally, the study quantified the expression of selected stress-associated genes to link molecular responses with observed physiological and agronomic performance. Overall, this research provides in-depth insights into the physio-chemical and molecular mechanisms underlying wheat productivity during the critical post-anthesis to grain-filling period under terminal heat stress.

## 2 Materials and methods

### 2.1 Plant material and growth condition

The field experiments were performed during two consecutive wheat-growing seasons, 2023–24 and 2024–25, at the research site (21°47′N, 39°43′E; altitude 270 m) of King Abdulaziz University, Jeddah, Saudi Arabia. The site is located in an arid climatic zone, with soil and metrological traits indicated in (**Supplementary Figures S1**, **S2**). A heat-sensitive bread wheat (*Triticum aestivum* L.) cultivar, HS-240 (Dhyani et al., 2013) was used as experimental material. The heat-sensitive wheat cultivar HS-240 was selected to ensure a pronounced and measurable stress response under elevated temperature conditions. The wheat cultivar ‘HS-240’ has been previously characterized as heat-sensitive, exhibiting a higher susceptibility index under during high temperature (Dhyani et al., 2013). Besides, the use of such heat-susceptible genotype allows clear expression of stress-induced physiological, biochemical, agronomic and molecular responses, which facilitates reliable evaluation of treatment effects under high temperature stress (Ullah et al., 2022). Seeds were surface-sterilized with 1% sodium hypochlorite for 5 minutes and rinsed thoroughly with distilled water. The seeds were sown manually in 2.5 m × 2.0 m plots at 25 cm row spacing, maintaining a seed rate of 120 kg ha^-^¹. Besides, the fertilizer was applied at 120 kg N, 90 kg P_2_O_5_, and 60 kg K_2_O ha^-^¹, split between sowing and tillering. Irrigation was applied through a drip system to maintain optimum soil moisture, and standard agronomic practices were performed uniformly throughout the growing seasons. Natural terminal heat stress was documented during the post-anthesis to grain-filling stages in both growing seasons, when mean daily maximum air temperatures consistently exceeded the optimal range of 22–25 °C for wheat growth. Heat stress intensity was quantified using standard thermal indices, including the number of heat-stress days (days with maximum temperature > 30 °C), cumulative exposure duration (total days above the threshold during grain filling), and heat degree days (HDD), calculated as the cumulative sum of daily maximum temperatures exceeding the 25 °C baseline during the grain-filling period (**Table 1**). Across both seasons, wheat plants experienced prolonged exposure to supra-optimal temperatures during grain filling, confirming the occurrence of natural terminal heat stress under field conditions. Accordingly, foliar treatments were applied at the heading stage (Feekes scale 10.3–10.5) to ensure that treatment effects were evaluated under well-characterized and reproducible heat-stress conditions.

**Table 1:** Terminal heat stress quantification during the grain-filling stages in wheat at Jeddah for 2023–24 and 2024–25 growing seasons.

Season	Grain filling period	Monthly avg. max temp (°C)	Heat stress days (>30 °C)	Cumulative days >30 °C	Heat degree days (Approx.)
2023–24	Apr–May 2024	Apr: 35.1; May: 37.0	60 days	60 days	300–350 °C days
2024–25	Apr–May 2025	Apr: 35.4; May: 37.3	60 days	60 days	305–355 °C days

### 2.2 Experimental design and treatments

**Figure 1:**
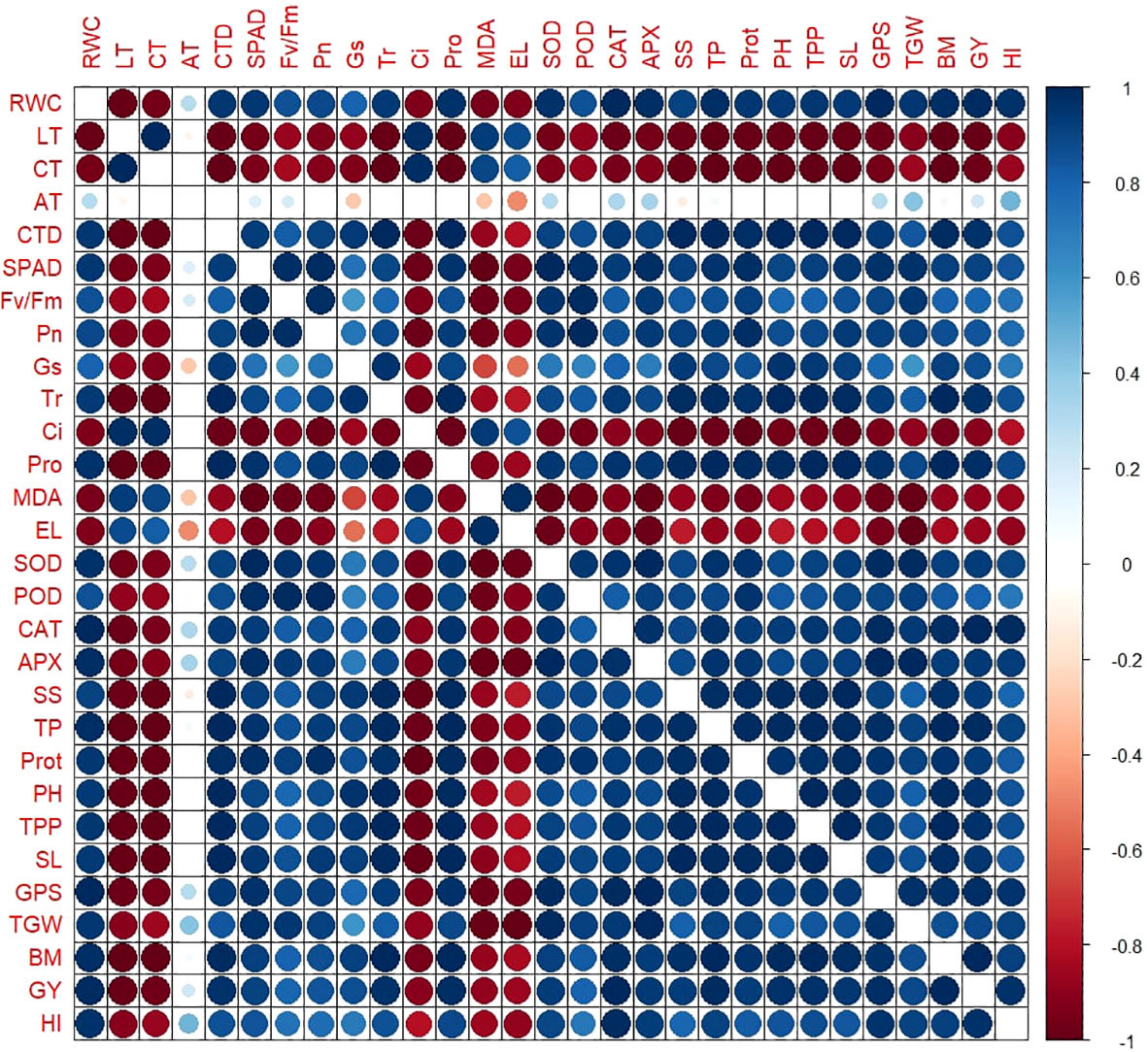
Correlation matrix among physiological, biochemical, and yield traits of heat stressed wheat plants under individual and combined foliar treatments. Positive correlations are shown in blue, while negative correlations are shown in red. The intensity and size of the circles correspond to the strength of the correlation coefficient (r). Mean data of two growing seasons (2023–24 and 2024–25) were used for analysis.

A randomized complete block design (RCBD) with three replicates was adopted for the experiment. Each treatment plot measured 2.5 m × 2.0 m with row spacing of 25 cm. Besides, buffer zones were maintained between plots to avoid spray interference. Four foliar spray treatments were applied at the heading stage (Feekes 10.3–10.5). These were: T0 (control, water spray), T1 (thiourea at 50 µM), T2 (KNO_3_ at 0.5% w/v), and T3 (thiourea 50 µM + KNO_3_ 0.5%). Spray solutions were prepared in distilled water with 0.05% along with Tween-20 as surfactant to improve adherence as used by Kaya et al. (2010). The solution pH was measured before application and maintained at approximately 6.5–6.8 to ensure thiourea stability. Foliar treatments were applied twice. Spraying was conducted in the morning between 08:30 and 10:30 a.m. during two consecutive wheat growing seasons (2023–2024 and 2024–2025) under comparable meteorological conditions, as shown in **Figure 1**. Sprays were applied using a knapsack sprayer having a fine-mist nozzle to ensure uniform coverage of the crop canopy (approximately 500 L ha^-^¹ spray volume). Foliar applications of thiourea and KNO_3_ were performed during the reproductive stage of wheat. The first spray was applied at Feekes growth stage 10.3 (Zadoks 55) when approximately 50% of the spikes had emerged from the flag leaf sheath, while the second foliar spray was applied 15 days after the first application, corresponding approximately to the early grain filling stage (Zadoks 70–71).

### 2.3 Physiological measurements

Physiological readings were taken from the fully expanded flag leaf at 3 and 7 days after treatment. RWC was determined using the method of Barrs and Weatherley (1962), by comparing fresh, turgid, and dry weights. Leaf (LT) and canopy temperatures (CT) were measured with an infrared thermometer ((Model MT4, Raytek Corporation, Santa Cruz, CA, USA), while ambient temperature (AT) was recorded simultaneously at canopy height. Canopy temperature depression (CTD) was calculated as the difference between AT and CT, following Reynolds et al. (1994). Canopy temperature was recorded using a handheld infrared thermometer between 12:00 and 14:00 h under clear sky conditions, opting the standard procedures for canopy temperature measurement in field-grown wheat as followed by Blum et al. (1982).

CTD was calculated as the difference between air temperature measured at canopy height (using a shaded, ventilated sensor) and canopy surface temperature measured with an infrared thermometer. Measurements were taken at the same time of day under comparable radiation and low-wind conditions across all treatments to minimize environmental variability. The relatively high CTD values observed reflect enhanced transpirational cooling under arid field conditions during terminal heat stress. Chlorophyll content was estimated using a SPAD-502 chlorophyll meter (Konica Minolta, Japan). Maximum photochemical efficiency of PSII (Fv/Fm) was measured after 30 min dark adaptation with a PAM fluorometer, as described by Maxwell and Johnson (2000). Gas exchange parameters, Pn, Gs, Tr, and intercellular CO_2_ concentration (Ci), were measured using a LI-6400XT portable photosynthesis system (LI-COR, USA), under fixed conditions of 400 µmol mol^-^¹ CO_2_ and saturating light intensity of 1200 µmol m^-^² s^-^¹, following the method opted by Long and Bernacchi (2003).

### 2.4 Biochemical analyses

For biochemical analysis flag leaves were collected 72 h after treatment, frozen in liquid nitrogen, and stored at −80 °C for analysis. The proline content was recorded using the acid-ninhydrin procedure of Bates et al. (1973). Besides, lipid peroxidation, expressed as malondialdehyde (MDA), was quantified using the thiobarbituric acid reaction (Heath and Packer, 1968). Electrolyte leakage (EL) was measured using the conductivity method explicated by Dionisio-Sese and Tobita (1998). Moreover, antioxidant enzymes were assayed from phosphate buffer extracts: superoxide dismutase (SOD) activity by NBT photoreduction inhibition (Giannopolitis and Ries, 1977); peroxidase (POD) activity using guaiacol oxidation (Chance and Maehly, 1955); catalase (CAT) activity by monitoring H_2_O_2_ decomposition at 240 nm (Aebi, 1984); and ascorbate peroxidase (APX) activity by measuring ascorbate oxidation (Nakano and Asada, 1981). Soluble sugars (SS) were recorded using the anthrone reagent method explained by (Yemm and Willis, 1954). Total phenolics were estimated with the Folin–Ciocalteu reagent assay (Singleton and Rossi, 1965). Total protein content was measured by following Bradford (1976) dye-binding method using bovine serum albumin as standard.

### 2.5 Yield and agronomic traits

At physiological maturity, three representative plants per plot were sampled for yield-related traits. To minimize border effects, the two outermost rows of each plot were excluded from sampling, and only plants from the central rows were considered. From each plot, three representative plants were randomly selected based on uniform growth and development, ensuring they accurately reflected the average performance of the plot. Plant height (PH) was measured from the soil surface to the tip of the spike. The number of tillers per plant, spike length (SL), and grains per spike (GPS) were manually counted. Thousand-grain weight (TGW) and grain yield (GY) per plant was determined using weighing balance. Above-ground biomass was recorded after drying at 70 °C to constant weight, and harvest index (HI) was calculated as grain yield divided by total biomass, expressed in percentage, following Donald and Hamblin (1976).

### 2.6 Statistical analysis

The experiment was conducted over two wheat growing seasons (2023–2024 and 2024–2025). Year was initially tested as a factor in the ANOVA, and the year × treatment interaction was examined. As neither the main effect of year nor the interaction was significant (P > 0.05), data from both seasons were pooled and averaged for final analysis. ANOVA assumptions were verified by testing residual normality and homogeneity of variances, and treatment means were compared using LSD at P ≤ 0.05. Moreover, correlation analysis among physiological, biochemical, and yield parameters was performed using Pearson’s coefficients. Besides, principal component analysis (PCA) and heatmap analysis was carried out to visualize multivariate treatment effects. All analyses were performed in R (version 4.3.2).

### 2.7 Gene expression analysis

Total RNA was extracted from frozen flag leaves samples using a plant RNA kit (Sigma-Aldrich, USA) with on-column DNase I treatment, and RNA quality was confirmed by spectrophotometry and agarose gel electrophoresis. Furthermore, cDNA was synthesized from 1 µg total RNA using the iScript cDNA Synthesis Kit (Bio-Rad Laboratories, USA; Cat. No. 1708891) as per the manufacturer’s protocol. Expression of ten heat-responsive genes (*TaHSP17, TaHSP90, TaDREB2, TaSOD, TaCAT, TaAPX, TaP5CS, TaGW2, TaCKX2*, and *TaSUS)* was quantified by SYBR (Thermo Fisher Scientific’s PowerUp, Cat# 4368814) Green-based qRT-PCR on a Bio-Rad CFX96 system using gene-specific primers. The reaction conditions were 95 °C for 3 min, followed by 40 cycles of 95 °C for 10 s and 60 °C for 30 s, with melt-curve analysis to validate the specificity. Reference gene TaACTIN was used for normalization after stability validation. Relative expression levels were calculated using the 2^−ΔΔCt method, with the control treatment as calibrator using three biological replicates and three technical replicates. The list of primers used for expression analysis is indicated in **Table 2**.

**Table 2:** List of Gene primers used in qRT-PCR analysis for relative expression analysis.

Gene	Forward primer (5’→3’)	Reverse primer (5’→3’)
TaHSP17	ATGGCTTCTTCCAGGTTTCT	TTAGCCACCTTGATGTTGGT
TaHSP90	AGCTTCACCTCAGGATCCAC	TCTTGGTGTTGTTGATGCGT
TaDREB2	GGTGATGTTGAGGAGGAGGA	CCACCTTGTCCTTGATGTCA
TaSOD	TGGTGACCTTCTCAGCTTTC	CCTTGGTGATGTTGCTCTTG
TaCAT	ATGGCTACTCCTCCTCCATC	TGGTGAGGTTGGTGATGTTG
TaAPX	GCTACTGGAAGGATGCTGAC	AGCCTTCTTGGTCTTCTTGC
TaP5CS	ATGACCATGGAGGAGTTTGC	CTTCTTGTCCTCCTCGTTGG
TaGW2	GGTGTTGAGCTGTTTGGTGT	CGGTTGTGGTAGTGTTGTTG
TaCKX2	GCTCTACCTCCTTGGTCTTG	TTCATCTTGGTCAGCTTGGT
TaSUS	ATGGTGGTGACTTTGCTGGT	TTCTTGACCTTGTTGGTGCT

## 3 Results

### 3.1 Physiological attributes

**Figure 2:**
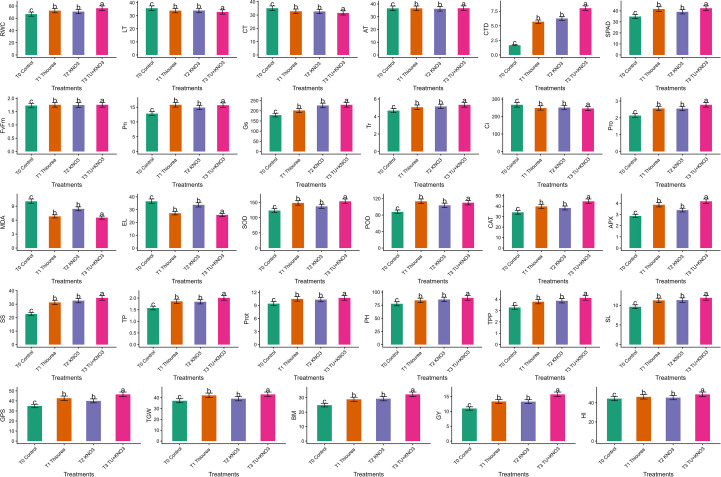
Effect of individual and combined treatments of thiourea (TU) and potassium nitrate (KNO_3_) on physiological, biochemical and yield related attributes of wheat under terminal heat stress. The parameters include relative water content (RWC, %), canopy temperature (CT, °C), leaf temperature (LT,°C), ambient temperature (AT, °C), canopy temperature depression (CTD), SPAD, photosynthesis (Pn, 8 µmol CO_2_ m^-^² s^-^¹), stomatal conductance (Gs, mmolm^-2^s^-1^), transpiration (Tr, mmol H_2_O m^-^² s^-^¹), photochemical efficiency of PSII (Fv/Fm), intercellular CO_2_ concentration (Ci, µmol mol^-^¹), total phenolics (TP, mg GAE g^-^¹ FW), soluble sugars (SS, mg g^-^¹ FW), ascorbate peroxidase (APX, U mg^-^¹ protein), catalase (CAT, U mg^-^¹ protein), peroxidase (POD, U mg^-^¹ protein), superoxide dismutase (SOD, U mg^-^¹ protein), electrolyte leakage (EL, %), malondialdehyde (MDA, nmol g^-^¹ FW), proline (µmol g^-^¹ FW), protein content (%), grain yield (GY, g), biomass (BM), thousand grain weight (TGW, g), grains per spike (GPS), spike length (SL, cm), plant height (PH, cm), harvest index (HI, %), and tillers per plant (TPP). Bars illustrate the mean of two growing seasons (2023–24 and 2024–25). Treatments include T_0_ (Control), T_1_ (Thiourea), T_2_ (KNO_3_), and T_3_ (Thiourea + KNO_3_). Different letters above bars represents significant differences among treatments at *p* ≤ 0.05 according to LSD test.

Exogenous application of thiourea (TU) and KNO_3_ significantly (p ≤ 0.05) improved physiological performance in wheat cultivar ‘HS-240’ compared to control (**Figure 2**). The photosynthetic rate (Pn) increased from 8.8 µmol CO_2_ m^-^² s^-^¹ in T0 (control, water spray) to 14.2 µmol CO_2_ m^-^² s^-^¹ in T_3_ (thiourea + KNO_3_), representing nearly a 60% improvement (**Figure 2**). The transpiration rate (Tr) increased slightly from 5.2 mmol H_2_O m^-^² s^-^¹ in T0 to 5.8 mmol H_2_O m^-^² s^-^¹ in T3, while SPAD values (chlorophyll content) rose significantly from 28 in T_0_ to 42 in T_3_ (**Figure 2**). The Fv/Fm ratio, an indicator of PSII efficiency, improved significantly (p ≤ 0.05) from 0.72 in T_0_ to 0.77 in T3 as indicated in **Figure 2**, confirming better photochemical activity under the combined application of TU and KNO_3_. Similarly, the rate of Gs was significantly (p ≤ 0.05) higher in T3 (260 mmolm^-2^s^-1^) compared with T_0_ (150 mmolm^-2^s^-1^) as mentioned in **Figure 2**.

Besides, all treatments significantly (p ≤ 0.05) affected the temperature related traits of heat stressed plants. The canopy temperature depression (CTD) increased from 2.5 °C in T0 to 9.0 °C in T3, while both canopy temperature (CT) and leaf temperature (LT) decreased from 35.5 °C and 36.0 °C in T0 to 30.0 °C and 32.0 °C in T3, respectively as indicated in **Figure 2**. This reduction indicated increased evaporative cooling and improved stomatal regulation. Moreover, the RWC increased significantly (p ≤ 0.05) from 65% in T_0_ to 83% in T_3_, confirming better water retention capacity as illustrated in **Figure 2**. In contrast, the intercellular CO_2_ concentration (Ci) declined significantly (p ≤ 0.05) from 270 µmol mol^-^¹ in T0 to 230 µmol mol^-^¹ in T3, reflecting improved CO_2_ assimilation efficiency (**Figure 2**). These physiological improvements indicate that both TU and KNO_3_ individually and combinedly protect photosynthetic machinery and water relations in heat stressed wheat plants, which provides the foundation for increased biochemical defense and agronomic performance.

### 3.2 Biochemical responses

The foliar application of TU and KNO_3_ significantly (p ≤ 0.05) affected all biochemical attributes in heat stressed ‘HS-240’ plant (**Figure 2**). The combined treatment (T3) recorded the highest accumulation of SS (38 mg g^-^¹ FW), proline (3.2 µmol g^-^¹ FW), and proteins (10%), compared with the respective control values of 22 mg g^-^¹ FW, 1.6 µmol g^-^¹ FW, and 6% in T0 as shown in **Figure 2**. Likewise, total phenolics increased significantly (p ≤ 0.05) from 16 mg GAE g^-^¹ FW in T0 to 22 mg in T3, indicating their role in stress regulation (**Figure 2**). Besides, the activity of antioxidant enzymes was significantly (p ≤ 0.05) higher in treated plants. The APX activity increased from 1.5 U mg^-^¹ protein in T_0_ to 3.9 U mg^-^¹ protein in T^3^, the CAT from 25 to 42 U mg^-^¹ protein, the POD from 70 to 105 U mg^-^¹ protein, and the SOD from 95 to 150 U mg^-^¹ protein as illustrated in **Figure 2**. This dramatic rise in the activities of antioxidant enzymes demonstrate the strong activation of the antioxidant defense system under TU and KNO_3_ application. Conversely the oxidative stress markers, the EL declined significantly (p ≤ 0.05) from 38% in T0 to 25% in T3, while MDA decreased significantly (p ≤ 0.05) from 10 nmol g^-^¹ FW to 6 nmol g^-^¹ FW as mentioned in **Figure 2**, indicating improved membrane stability and reduced lipid peroxidation. Overall, these biochemical changes further endorse the physiological findings, as osmoprotectant accumulation and antioxidant activation synergistically safeguard photosynthetic machinery, water balance, which in turn support better growth and yield performance of heat susceptible cultivar ‘HS-240’ under heat stress.

### 3.3 Agronomic traits

Both individual and combined treatments of TU and KNO_3_ significantly (p ≤ 0.05) improved agronomic traits and yield performance in heat stressed ‘HS-240’ plants (**Figure 2**). The GY increased significantly (p ≤ 0.05) from 12 g plant^-^¹ in T0 to 16 g in T3, while BM improved from 24 g (T0) to 34 g (T3) as indicated in **Figure 2**. In the same way, TGW increased from 34 g in T0 to 42 g in T3, and GPS rose from 32 in T0 to 46 in T3 as shown in **Figure 2**. The combined treatment also significantly (p ≤ 0.05) enhanced SL (9.5 to 12.5 cm), PH (70 to 88 cm), and TPP (2.8 to 4.2) as indicated in **Figure 2**. Moreover, the HI increased from 43% in T0 to 49% in T3, indicating more efficient partitioning of assimilates into the grain (**Figure 2**). Overall, the agronomic improvements are the collective outcome of better physiological stability and biochemical defense, enabling plants to allocate resources more efficiently to yield components in heat stressed wheat cultivar ‘HS-240’.

### 3.4 Correlation matrix and PCA-biplot

The correlation matrix demonstrated a highly structured association between physiological, biochemical, and yield-related agronomic traits in heat stressed wheat cultivar ‘HS-240’ (**Figure 1**). The physiological traits (RWC, SPAD values, Pn, Gs), antioxidant enzymes (SOD, POD, CAT, APX), and agronomic traits (GY, TGW, HI) correlated positively and varied in same direction (**Figure 1**). On the other hand, EL and MDA, showed strong paired association in opposite direction with photosynthetic traits (SPAD, Pn, Ci, Gs) and agronomic yield traits (TGW, SL, TPP, HI, GY), highlighting their role as indicators of tissues injury and oxidative stress (**Figure 1**).

**Figure 3:**
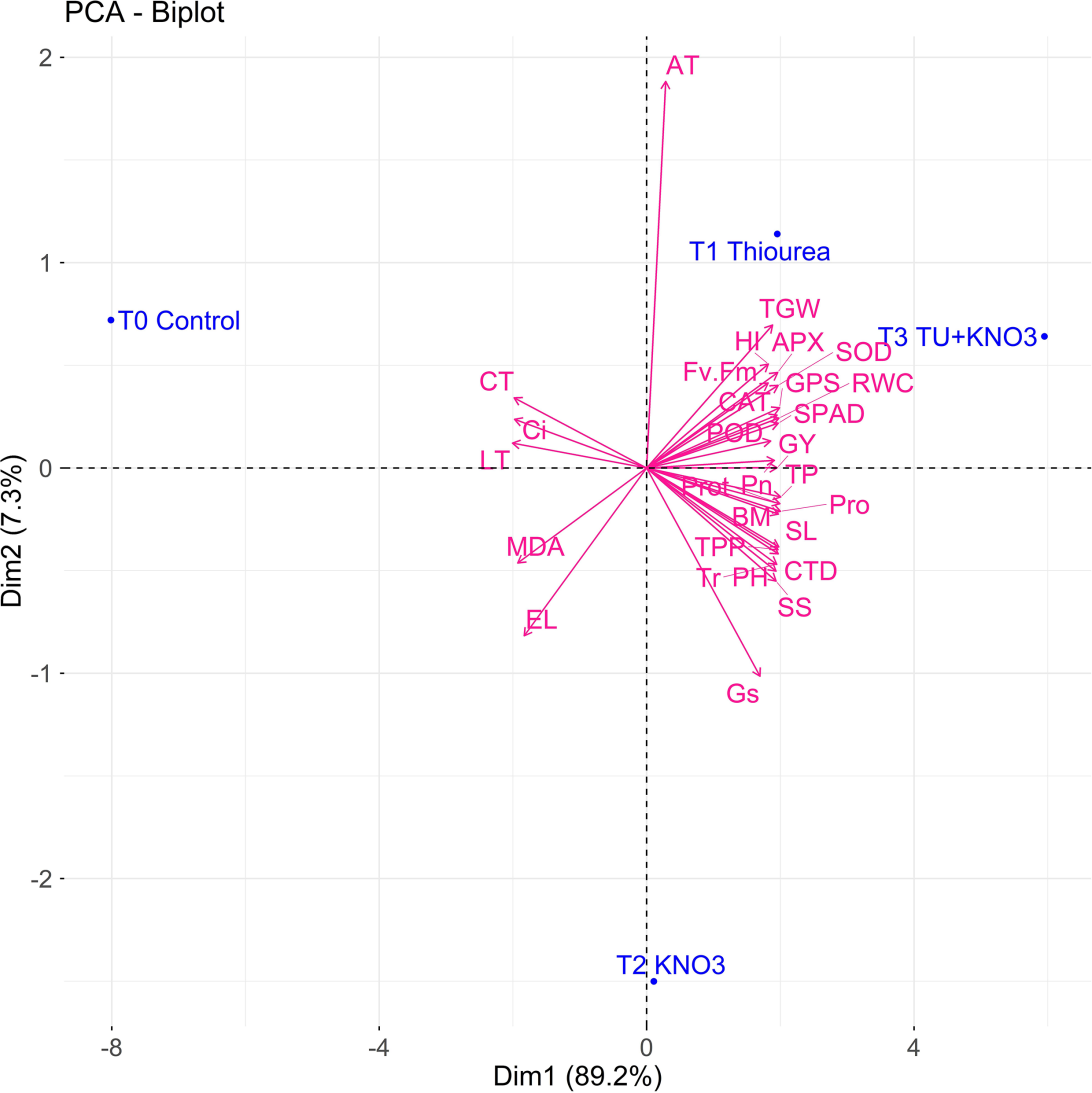
Principal component analysis (PCA) biplot expressing associations among physiological, biochemical, and yield traits and their response to different treatments. Treatments include T_0_ (Control), T_1_ (Thiourea), T_2_ (KNO_3_), and T_3_ (Thiourea + KNO_3_). The first two principal components (Dim1 and Dim2) express 89.2% and 7.3% of total variation, respectively. Mean data of two growing seasons (2023–24 and 2024–25) were used for PCA.

The PCA-biplot illustrated 89.2% (Dim1) and 7.3% (Dim2) of the total variance, showing distinct separation of treatments as illustrated in **Figure 3**. The control (T_0_) was clearly separated from both individual and combined foliar treatments (T1, T2 and T3), aligning closer to stress injury markers MDA, and EL (**Figure 3**). In contrast, T1 grouped closer to proline and SS as indicated in PCA **Figure 3**, highlighting the role of TU in osmolyte-mediated protection and chlorophyll maintenance. Besides, T_2_ spaced closely with antioxidative and traits (**Figure 3**), confirming the potential role of KNO_3_ in ROS detoxification and photosynthetic stability and. Importantly, T_3_ (TU + KNO_3_) aligned with both physio-chemical and agronomic traits, occupying an intermediate but broader position in **Figure 3** which represents a collective response that integrates osmolyte accumulation, antioxidative defense, and photosynthetic stabilization. These clustering patterns (**Figure 3**) confirms the target specific effect of individual treatments and broad-spectrum effects of combined treatments.

### 3.5 PCA eclipses and heatmap of trait responses

**Figure 4:**
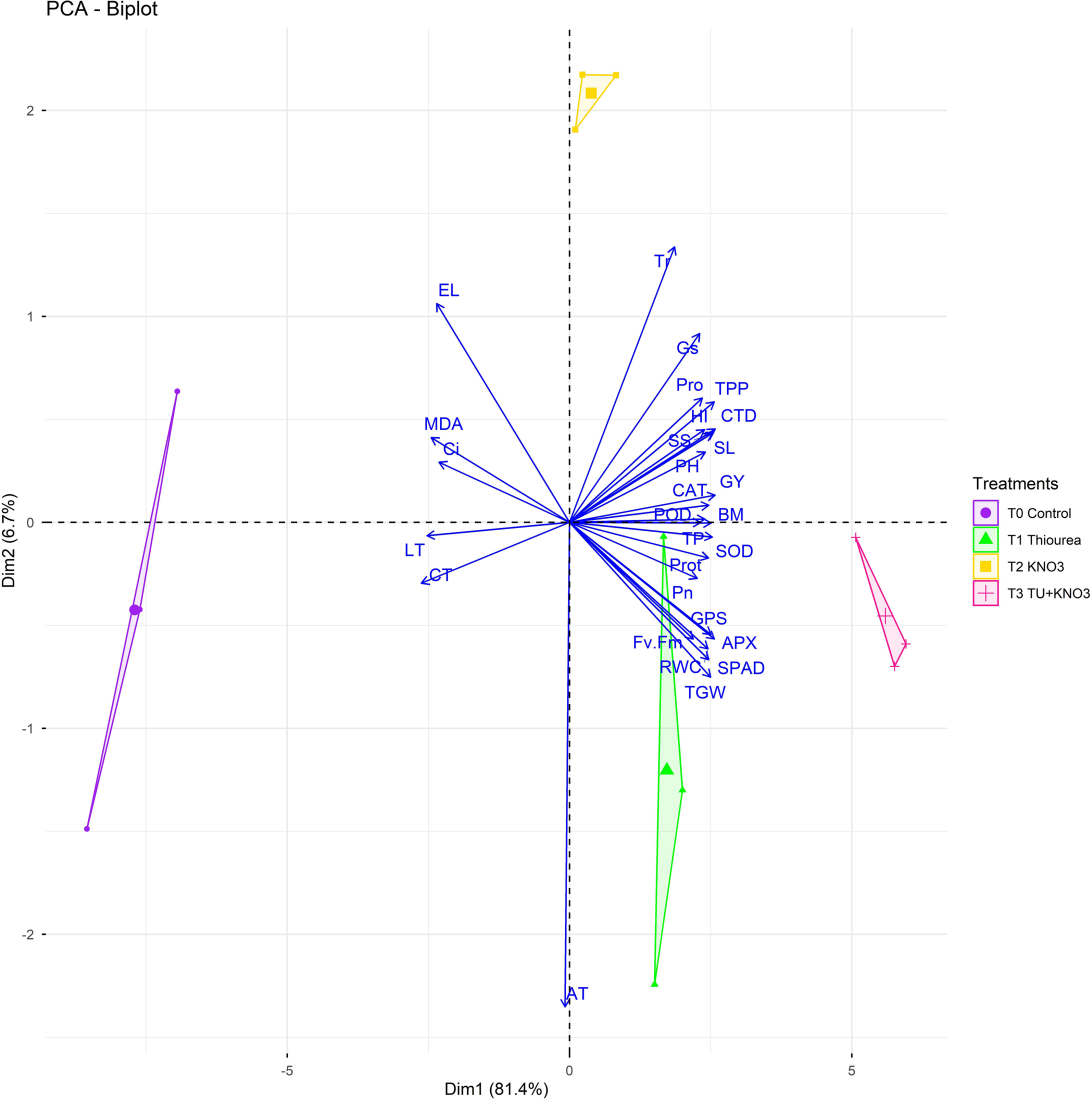
Principal component analysis (PCA) biplot representing treatment clustering and trait associations under terminal heat stress conditions. Treatments (T_0_–T_3_) are represented by distinct symbols and colors. The first two components (Dim1 = 81.4%, Dim2 = 6.7%) collectively explain the majority of variation among traits. Data show means of the two wheat growing seasons (2023–24 and 2024–25).

**Figure 5:**
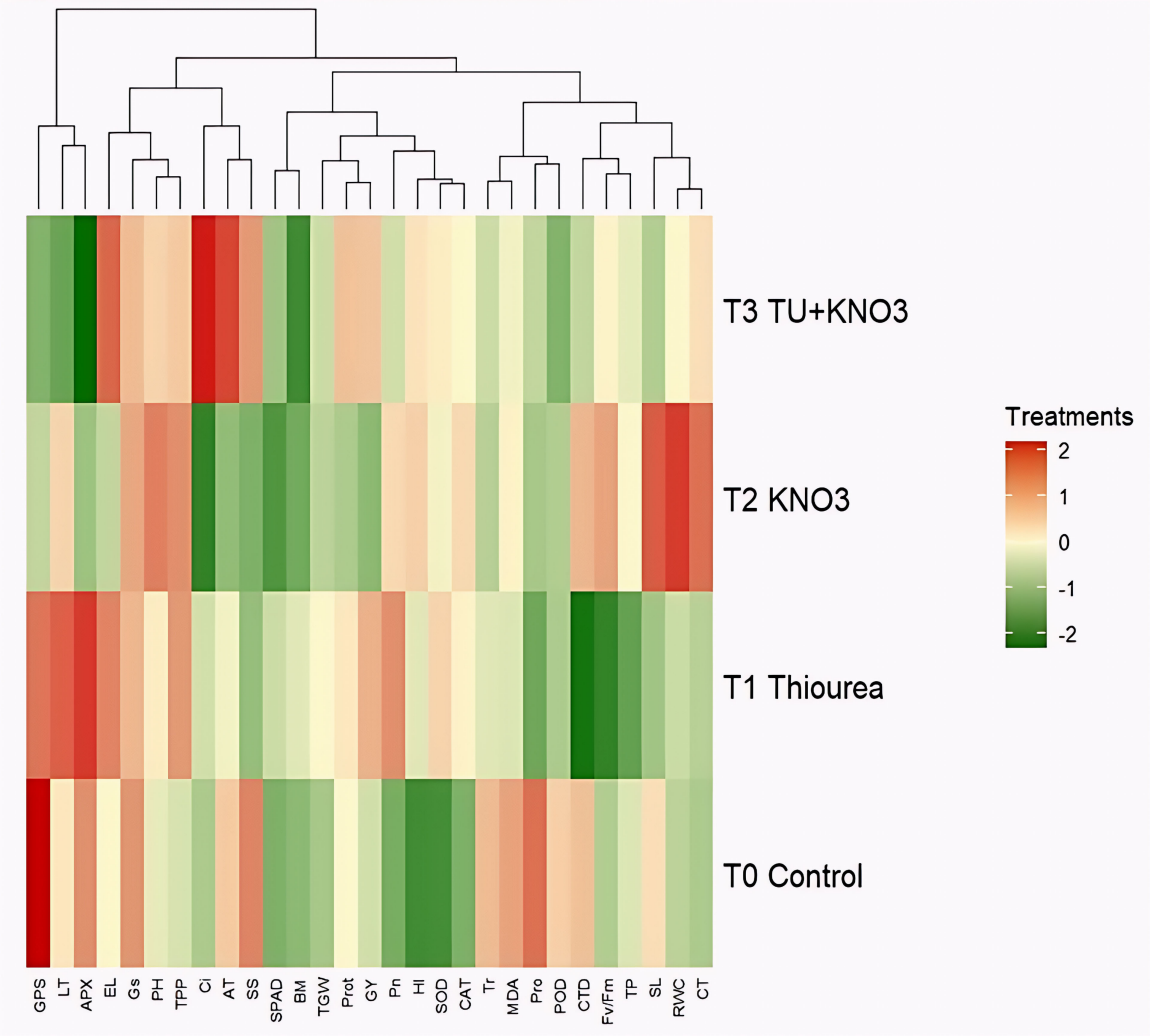
Hierarchical clustering heatmap representing the response physiological, biochemical and yield traits of wheat to different foliar treatments under terminal heat stress. Treatments include T_0_ (Control), T_1_ (Thiourea), T_2_ (KNO_3_), and T_3_ (Thiourea + KNO_3_). Red shades show higher trait expression, while green shades indicate lower expression levels. Data are based on the mean of two field seasons (2023–24 and 2024–25).

The second PCA biplot from **Figure 4** illustrated treatment specific clustering pattern and accounted for 81.4% (Dim1) and 6.7% (Dim2) of variation. T_0_ (control) spaced distantly from physiological and agronomic traits, reflecting susceptibility to stress (**Figure 4**). In contrast, T2 (KNO_3_) and T3 (TU + KNO_3_) were strongly associated with RWC, SPAD, Pn, GY, TGW, and antioxidant enzymes (APX, POD, SOD, CAT), indicating superior tolerance to heat stress (**Figure 4**). Besides, TU (T1), while associated with osmolyte accumulation (proline, SS) and chlorophyll stabilization, showed comparatively suppressed effect on yield traits compared to KNO_3_ or the combined treatment (**Figure 4**). Besides, the hierarchical clustering heatmap further verified these associations, clearly differentiating stress-mitigating treatments from the control (**Figure 5**). Moreover, T3 depicted the superior trait profile, with higher values of RWC, Pn, SPAD, antioxidant enzymes, and yield components, while simultaneously exhibiting the lowest levels of stress indicators MDA, EL and LT as mentioned in **Figure 5**. Besides, in heatmap clustering T2 followed closely, particularly in its strong antioxidant activity, whereas T1 showed moderate but stable improvement in osmolyte, Pn and chlorophyll-related traits (**Figure 5**). The control plants clustered separately, with elevated stress injury markers with low expression of physiological and agronomic traits (**Figure 5**). Overall, these multivariate analyses confirmed the complementary roles of TU and KNO_3_, with their combined application providing the most comprehensive mitigation of oxidative stress and improvement in growth and productivity.

### 3.6 Relative gene expression and heatmap of heat stress-associated genes

**Figure 6:**
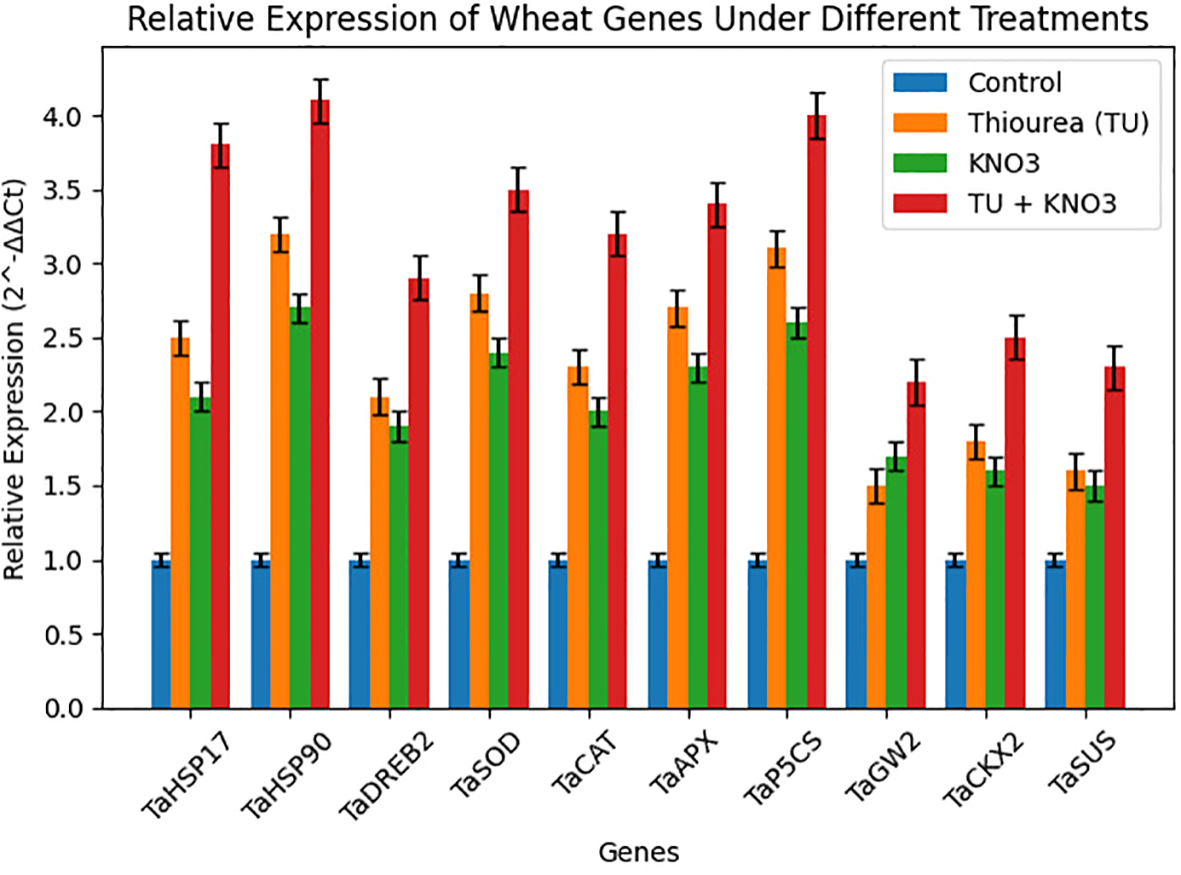
Relative expression of heat-stress responsive genes in wheat during terminal heat stress under different foliar treatments during 2023–24 and 2024–25. Treatments include T_0_ (Control), T_1_ (Thiourea), T_2_ (KNO_3_), and T_3_ (Thiourea + KNO_3_). Gene expression was quantified using qPCR and normalized to the reference gene [Reference Gene]. Data represent mean ± SE (n = 3). Different letters above the bars indicate significant differences among treatments (p < 0.05) based on one-way ANOVA followed by Tukey’s HSD *post hoc* test.

The relative expression profile of stress associated genes revealed a clear and treatment-dependent modulation of in heat stressed wheat plant as shown in **Figure 6**. The studied set of heat shock proteins (*TaHSP17, TaHSP90*), antioxidant enzymes (*TaSOD, TaCAT, TaAPX*), osmolyte regulatory genes (*TaP5CS*), carbohydrate metabolism genes (*TaSUS*), yield-regulators (*TaGW2, TaCKX2*), and transcription factors (*TaDREB2*), indicated significant (p ≤ 0.05) increase in expression under foliar treatments of osmoprotectants compared to the control (**Figure 6**). The combined T3 (TU + KNO_3_) treatment demonstrated the significant upregulation of *TaHSP90* (4.1-fold), *TaP5CS* (4.0-fold), and *TaHSP17* (3.8-fold). These genetic responses were aligned with improved RWC, higher SPAD values, sustained Pn and enhanced proline (**Figures 6**, **2**). Correspondingly, the integrated application of TU and KNO_3_ significantly (p ≤ 0.05) upregulated *TaAPX, TaSOD*, and *TaCAT* (2.3–2.8 fold) that was consistent with enhanced antioxidant activities, lower MDA, and reduced EL (**Figures 2**, **6**). Moreover, *TaDREB2* expression was consistently higher under T2 and T3 (**Figure 6**), which corresponded to improved Gs, Pn, and osmotic adjustment (**Figure 2**). Beyond stress protection, the foliar treatments of osmoprotectants also influenced yield-related genes. For instance, *TaGW2* and *TaSUS* showed moderate but significant (p ≤ 0.05) upregulation under combined treatment T_3_ (**Figure 6**), aligning with improved TGW and grain filling under T3 treatment as shown in **Figure 2**. Correspondingly, *TaCKX2*, a cytokinin oxidase gene illustrated significant (p ≤ 0.05) upregulation in heat stressed wheat plants under T_3_ (**Figure 6**), correlating with better HI and GY, as higher sink activity supported assimilate partitioning to reproductive organs.

**Figure 7:**
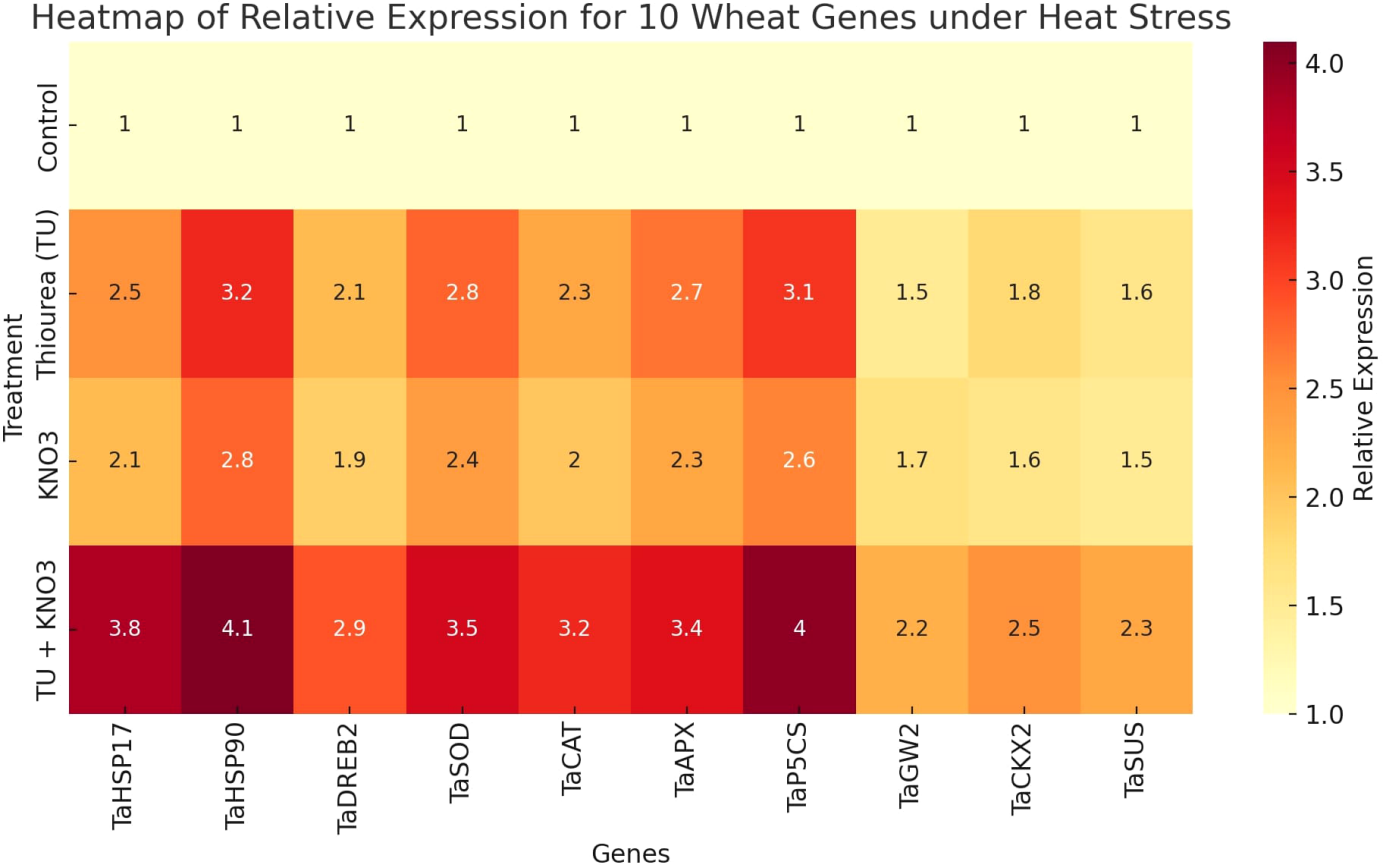
Heatmap of relative gene expression for ten heat-stress responsive wheat genes under different foliar treatments. Treatments include T_0_ (Control), T_1_ (Thiourea), T_2_ (KNO_3_), and T_3_ (Thiourea + KNO_3_).

The expression heatmap also displayed a clear difference in the expression genes due to foliar application of osmoprotectants (**Figure 7**). Here, T3 clustered at the highest expression level across nearly all genes, followed by T2, T1, and T0 (**Figure 7**). This expression heatmap further verified the outcomes of gene expression in **Figure 6**. It reflects that TU and KNO_3_ treatments not only mitigates stress but also modulate transcriptional activity to enhance yield stability in heat stressed wheat cultivar. Overall, these gene-level responses (**Figures 6**, **7**) coincided closely with physiological improvements (RWC, SPAD, Pn, Gs), biochemical attributes (antioxidant activity, reduced MDA and EL), and agronomic indices (GY, TGW, HI), hence creating a strong functional link between molecular determinants and whole-plant performance.

## 4 Discussion

The present study found that foliar treatments of osmoprotectants, TU and KNO_3_ is an effective method to alleviate heat stress in wheat, with the combined treatment T3 (TU + KNO_3_) consistently surpassing individual treatments. Physiological responses of heat stressed plants indicated that T3 significantly (p ≤ 0.05) enhanced RWC, SPAD index, Pn, and Gs, while reducing CT and LT compared with the untreated plants (**Figure 2**). These improvements suggest that the integrated application KNO_3_ and TU optimized stomatal functioning and sustained plant water relations in heat stressed plants, thereby maintained the carbon assimilation (**Figure 2**). Caverzan et al. (2016) and Hasanuzzaman et al. (2020) reported similar findings and concluded that exogenous osmoprotectants safeguard photosynthetic machinery by reducing oxidative load and stabilizing the membrane systems. Moreover, Khan et al. (2021) and Ahmad et al. (2024) further endorsed the findings from present study and inferred that nitrates and TU stabilize chloroplast ultrastructure and sustain physiological processes in heat-affected cereals during terminal growth stages. In addition to physiological findings (**Figure 2**), the complementary effect of TU and KNO_3_ was further ratified from biochemical findings (**Figure 2**). The combined treatment of osmoprotectants increased osmolytic concentration of proline, GB, SS, and phenolics, and enhanced the antioxidant activities of SOD, CAT, POD, and APX, and lowered MDA and EL (**Figure 2**), that indicated minimized lipid peroxidation and membrane destabilization in heat stressed wheat plants. Besides, these broad mechanisms of osmotic adjustment and ROS detoxification confirmed the broader protection conferred by TU and KNO_3_. Correspondingly, the stress mitigating effects of TU and KNO3 in improving terminal heat tolerance of wheat by improving antioxidants and osmolytes were documented in wheat by Ahmad et al. (2021), Wang X. et al. (2023) and Qiao et al. (2025). In addition, solutes like proline and GB have been widely reported to enhance osmotic adjustment and increasing ROS scavenging tendency (Hasanuzzaman et al., 2014). The observed rise of phenolic compounds further confirms their role in antioxidative defense and signaling under heat stress as supported by the findings of Karamzadeh et al. (2025).

Furthermore, the agronomic yield correlated with physiological and biochemical improvements. The combined treatment (T3) recorded superior biomass, TGW, GY, and HI in heat stressed wheat plant compared with individual applications or control (**Figure 2**). These improvements assured that stress alleviation at physiological stages translates into stable agronomic yield. Besides, the correlation matrix (**Figure 1**) depicted strong positive paired associations of RWC, SPAD, Pn, proline, and antioxidant enzyme activities, with yield components GY and TGW. However, MDA and EL were negatively correlated with yield components. These results align with earlier studies that associated higher membrane stability and antioxidant activity to yield protection under heat stress in wheat (Farooq et al., 2011; Kumar et al., 2017; Janni et al., 2020). Previous reports also confirm that osmoprotectant treatments and enhanced antioxidant activities increase assimilate partitioning efficiency and grain filling, which results in higher HI under heat stress (Suryavanshi et al., 2016; Farooq, 2023).

Furthermore, principal component analysis (PCA) and clustering (**Figures 3**, **4**) clearly separated treatments based on integrated physiological and biochemical profiles. T_3_ clustered with higher values for RWC, SPAD, Pn, proline, and antioxidant activities, while T2 (KNO_3_) aligned more with antioxidant traits and T1 (TU) with osmolyte-related traits. This distribution highlights that the combined treatment provided broader and more balanced protection compared with individual treatments. Such multivariate separation of treatments has been reported in wheat and rice under heat and drought stress, where combined biochemical, physiological adaptations lead to distinct clustering of stress-protected plants (Rehman et al., 2019; Zhang et al., 2021). Gene expression profiling (**Figure 6**) provided further in-depth insight into stress adaptation. The ten heat-responsive genes including heat shock proteins (*TaHSP17, TaHSP90*), antioxidant enzyme genes (*TaSOD, TaCAT, TaAPX*), osmolyte biosynthesis gene (*TaP5CS*), transcription factor (*TaDREB2*), and yield-related regulators (*TaSUS, TaGW2, TaCKX*2) were evaluated for their expression in present study. The combined treatment (T3) strongly induced *TaHSP90* (4.1-fold), *TaP5CS* (4.0-fold), and *TaHSP17* (3.8-fold), reflecting enhanced protein stabilization, osmolyte accumulation, and protection under heat stress. This expression pattern corresponds with higher SPAD, improved Fv/Fm, and elevated proline levels, establishing a direct molecular-to-physiological link. These results were parallel with the research outcomes of Wang Y. et al. (2023) and Li et al. (2023), who established the role of *TaHSPs* and *TaP5CS* in protecting the physiological machinery of wheat during terminal heat stress. Similarly, T_3_ preferentially upregulated *TaSOD, TaCAT*, and *TaAPX* (2.3–2.8 fold), which paralleled increased antioxidant activities and reduced oxidative markers (MDA, EL), confirming its role in heat induced redox stress regulation. These results were further confirmed by Ahmad et al. (2024), who reported the upregulation of antioxidant systems along with oxidative markers in heat stressed *Brassica napus* under the foliar supplementation of osmoprotectants, *TaDREB2* was induced in both T_2_ and T_3_, supporting its role in transcriptional control of osmotic and antioxidant pathways, consistent with DREB-mediated stress responses in wheat as reported by Qin et al. (2020) and Alshareef et al. (2022). Besides, yield-associated genes also showed treatment-specific regulation during heat stress. *TaSUS* was moderately upregulated in heat stresses wheat plant under T3, supporting enhanced sucrose metabolism and carbon partitioning, while *TaGW2* expression indicated a role in grain size determination and improved TGW under combined treatment application. *TaCKX2*, which control cytokinin metabolism and sink activity, was enhanced under T3, corresponding with higher HI and grain yield. These results confirm that the transcriptional regulation induced by TU + KNO_3_ not only impart protection against heat stress but also regulate the yield stability pathways. Similar correlations between source and sink regulation genes and yield stability under stress have been observed by Yang et al. (2020) and Waqas et al. (2022).

The gene expression heatmap shown in **Figure 7** has clearly separated treatments, with T3 clustering at the highest expression level across all 10 genes, followed by T2, T1, and control. This hierarchical clustering was in complete agreement with the physiological and agronomic outcomes described in **Figure 2**, confirming that gene-level activation reinforced trait-level improvements. The coherence across molecular, biochemical, physiological, and agronomic indicators of heat stress tolerance suggests that TU and KNO_3_ supplementation orchestrates multi-level regulatory mechanisms to ensure heat tolerance in wheat.

Since a single wheat genotype was evaluated in present study, the findings focus on the genotype-specific transcriptional plasticity that allows integration of osmotic adjustment, antioxidant regulation, protein stabilization, and yield stability under heat stress. Although this may limit broad scale generalization; however, it provides a precise model that can guide genotype screening and breeding strategies. The integration of TU and KNO_3_ appears particularly effective in synchronizing stress tolerance pathways, supporting earlier claims that combined nutrient and protectant applications outperform individual treatments under climate stress (Ashraf and Harris, 2013; Hasanuzzaman et al., 2013; Waqas et al., 2022).

Collectively, the integration of physiological (**Figure 2**), biochemical (**Figure 2**), agronomic (**Figure 2**), correlation and multivariate analyses (**Figures 1**-**5**), and gene-expression profiling (Fig) provides clear evidence that integrated supplementation of TU + KNO_3_ confers strong heat stress resilience in heat susceptible wheat cultivar ‘HS-240’. These results indicate the potential of TU and KNO_3_ foliar sprays as practical and cost-effective agronomic measure to sustain wheat productivity under rising global temperatures. Furthermore, the use of a highly susceptible genotype enabled a clearer evaluation of heat-induced physiological, biochemical, and molecular alterations, as well as the mitigation potential of the applied treatment under severe stress conditions. Although inclusion of a heat-tolerant cultivar would have provided comparative insights into genotype-specific heat response mechanisms, the present study was designed as a focused mechanistic investigation using a single heat-sensitive genotype. Future studies incorporating contrasting heat-tolerant and heat-sensitive cultivars would further strengthen understanding of differential stress adaptation strategies.

## Data Availability

The original contributions presented in the study are included in the article/**Supplementary Material**. Further inquiries can be directed to the corresponding author.

## Supplementary material

Supplementary Figure 1 Location and soil characteristics of the experimental site (Hada Al Sham). The map represents the geographical position of the experimental site in the western part of Saudi Arabia, marked within the Makkah Province. The inset displays the location of Hada Al Sham (in pink) and its corresponding soil characteristics. The soil textural class was sandy loam having pH of 7.8 and an electrical conductivity (EC) of 2.2 dS m^-^¹.

Supplementary Figure 2 Monthly climatic variations at Hada Al Sham research station during the 2023–24 and 2024–25 wheat growing seasons. The graphs indicate the average temperature (°C), relative humidity (%), wind speed (km h^-^¹), and rainfall (mm) in the growing months (December to May). Data were obtained from the local meteorological station, and mean values of the two seasons were used for graphical presentation.
