# Supplementary figures and images for: Foliar thiourea and potassium nitrate enhance physiological performance, antioxidant defense, and yield of heat-stressed wheat under field conditions
Source: Front Plant Sci. 2026 Apr 10;17:1766062. doi: 10.3389/fpls.2026.1766062 (PMC13108340; doi:10.3389/fpls.2026.1766062)

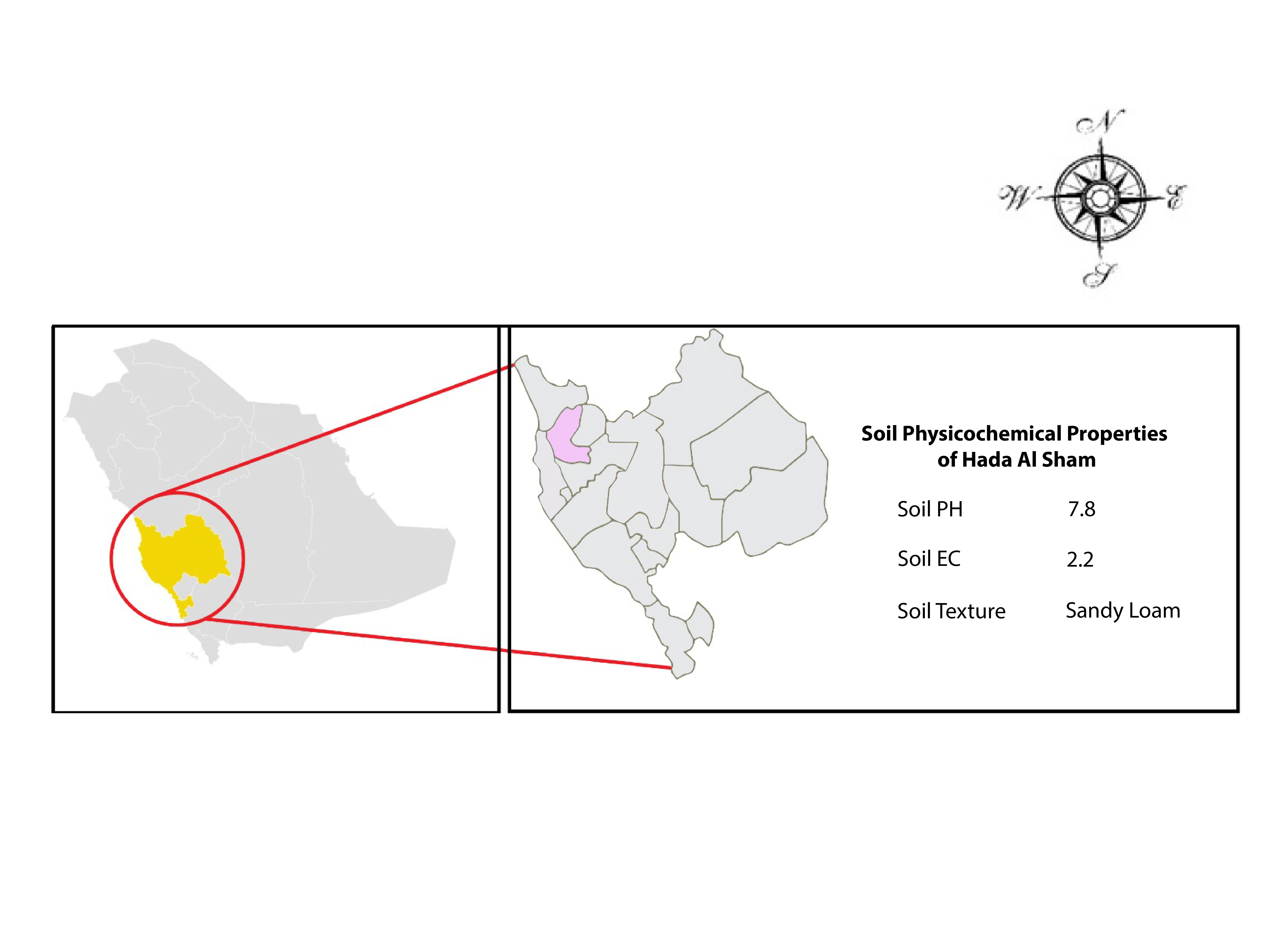

Supplement: Supplementary Figure 1 — Location and soil characteristics of the experimental site (Hada Al Sham). The map represents the geographical position of the experimental site in the western part of Saudi Arabia, marked within the Makkah Province. The inset displays the location of Hada Al Sham (in pink) and its corresponding soil characteristics. The soil textural class was sandy loam having pH of 7.8 and an electrical conductivity (EC) of 2.2 dS m-¹. [file Image1.jpeg]
